# Clinical consequences of delayed recognition of gastrointestinal basidiobolomycosis: a case series

**DOI:** 10.1093/jscr/rjag201

**Published:** 2026-03-26

**Authors:** Aram Almasaud, Saeed Alghamdi, Ali Alzahrani, Rami Sairafi, Lara Alkhelaiwy, Ammar Haidari

**Affiliations:** Department of General Surgery, Security Forces Hospital, Riyadh 11481, Saudi Arabia; Department of General Surgery, Security Forces Hospital, Riyadh 11481, Saudi Arabia; Department of Colorectal Surgery, Security Forces Hospital, Riyadh 11481, Saudi Arabia; Department of Colorectal Surgery, Security Forces Hospital, Riyadh 11481, Saudi Arabia; Department of General Surgery, Specialized Medical Center, Riyadh 11481, Saudi Arabia; Department of General Surgery, King Faisal Specialist Hospital and Research Centre, Riyadh 11481, Saudi Arabia

**Keywords:** gastrointestinal basidiobolomycosis, *Basidiobolus ranarum*, fungal colitis, inflammatory bowel disease mimic, voriconazole therapy

## Abstract

Gastrointestinal basidiobolomycosis (GIB) is a rare infection that often mimics colorectal malignancy or inflammatory bowel disease, leading to delayed diagnosis. A 20-year-old man presented with acute right iliac fossa pain and imaging suggestive of a cecal carcinoma with hepatic metastases. Colonoscopy showed a large fungating cecal mass, and biopsies demonstrated necrotizing granulomatous inflammation with broad pauciseptate hyphae compatible with basidiobolomycosis. A 14-year-old girl had mucous diarrhea, significant weight loss and was labeled as ulcerative colitis despite non-diagnostic histology, then developed mesalazine-related hypokalemia with cardiac arrest. Abdominal magnetic resonance imaging at a tertiary centre showed severe rectosigmoid colitis with peritoneal disease, and omental biopsy confirmed GIB. Both patients were treated with prolonged voriconazole. The first had complete clinical and radiologic resolution of colonic and hepatic disease without surgery. The second had marked regression of rectosigmoid and peritoneal disease, with later radiologic recurrence of pelvic disease despite ongoing therapy, but remains clinically stable under multidisciplinary follow-up. This series highlights GIB as an important differential diagnosis for refractory colitis in immunocompetent patients and supports the use of voriconazole as an organ-sparing option when the diagnosis is made before complications develop.

## Introduction

*Basidiobolus ranarum* is an environmental saprophytic fungus of the order Entomophthorales (class Zygomycetes) that inhabits soil, decaying plant material, and the feces of amphibians, reptiles, and insectivorous animals [1, 2]. Human infection, termed basidiobolomycosis, has been reported predominantly from tropical and subtropical regions, including parts of the Middle East, Africa, South America, and the southern United States [3, 4]. Classically, basidiobolomycosis is a chronic subcutaneous infection affecting otherwise healthy individuals, characterized by firm, painless induration of the limbs, trunk, or buttocks [5]. Minor trauma, insect bites, and local inoculation are thought to be the main routes of entry [2, 5].

Over the last two decades, extracutaneous disease has been increasingly recognized. Gastrointestinal basidiobolomycosis (GIB) has emerged as an uncommon but essential infection involving the stomach, small intestine, colon, and, less frequently, the liver or peritoneum [2, 4]. Most reported patients are immunocompetent children or young adults living in or with exposure to endemic areas [6, 7]. The clinical picture is non-specific and may include fever, abdominal pain, chronic diarrhea, weight loss, abdominal mass, or features of bowel obstruction [4, 7–9]. Because of its mass-forming and transmural nature, GIB often mimics more common conditions such as colorectal or small bowel malignancy, lymphoma, appendicitis, inflammatory bowel disease, and intestinal tuberculosis [1].

Early diagnosis is challenging. Symptoms and imaging findings lack specificity, and routine laboratory markers are not diagnostic, although leukocytosis, eosinophilia, and elevated inflammatory markers are frequently observed [4, 6]. Superficial endoscopic biopsies may be non-representative, as the lesion typically involves the submucosa and deeper layers [3, 10]. Definitive diagnosis usually relies on histopathological examination of adequately sampled tissue showing granulomatous inflammation with broad, pauciseptate fungal hyphae consistent with *B. ranarum*, supported by culture or molecular methods when available [3, 11]. Failure to recognize GIB can lead to inappropriate immunosuppressive or oncologic therapies, unnecessary delay in antifungal treatment, and avoidable complications such as perforation, abscess formation, or death [12].

We report two cases of GIB with distinct intra-abdominal manifestations managed at a single tertiary center. The series illustrates the broad clinical spectrum of GIB, the potential for misdiagnosis as an inflammatory or malignant disease, and the pivotal roles of histopathology and multidisciplinary decision-making in guiding timely antifungal therapy and, when required, surgical intervention.

## Case presentation

### Case 1

A 20-year-old previously healthy male presented to the emergency department with a 3-day history of right iliac fossa pain. The pain was localized, non-radiating, and not associated with fever, nausea, vomiting, altered bowel habits, weight loss, or previous abdominal complaints. On examination, he was hemodynamically stable; the abdomen was soft with mild tenderness in the right lower quadrant and no guarding, rebound tenderness, or palpable mass. Baseline laboratory investigations were non-specific and did not suggest an alternative diagnosis.

**Figure 1:**
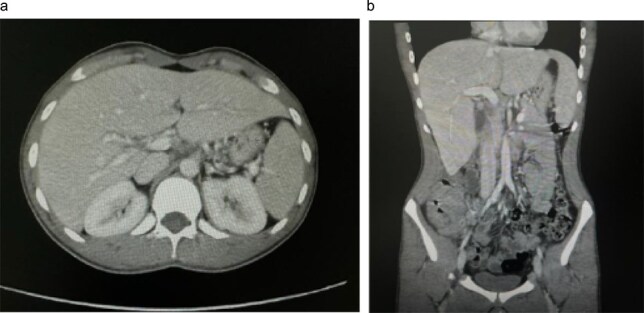
Initial contrast-enhanced computed tomography (CT) abdomen. (a) Axial image demonstrating a 3.8 × 3.3 cm lesion in hepatic segment 4B, suspicious for metastasis. (b) Coronal reformatted image showing the hepatic lesion in association with right-sided colonic wall thickening, raising initial concern for primary colonic malignancy with hepatic metastasis.

Initial contrast-enhanced computerized tomography scan of the abdomen demonstrated circumferential cecal wall thickening measuring 5.8 × 2.6 cm, a dilated appendix, and a 3.8 × 3.3 cm lesion in hepatic segment 4B, findings concerning for a right-sided colonic malignancy with hepatic metastasis. Magnetic resonance imaging (MRI) confirmed two atypical liver lesions in segments 4B and 6/7, which, in the context of right-sided colonic thickening, were considered highly suspicious for metastatic disease; biopsy of the segment 4B lesion was recommended if the colonic thickening proved non-neoplastic. Colonoscopy revealed a large fungating cecal mass (Fig. 1). Histopathological examination of biopsies demonstrated necrotizing granulomatous inflammation with fungal organisms consistent with basidiobolomycosis, and repeated biopsies confirmed the diagnosis of GIB with hepatic involvement.

**Figure 2:**
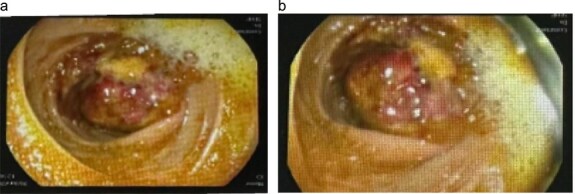
Colonoscopic appearance of the cecal lesion (a, b). Endoscopic views showing a large fungating, circumferential cecal mass with ulcerated, erythematous mucosa, and adherent yellowish exudate causing near-luminal occlusion; biopsies confirmed GIB due to *Basidiobolus ranarum.*

Following multidisciplinary team discussion, the patient was managed medically with voriconazole, administered at 400 mg every 12 h for one day, then 200 mg every 12 h for a total of 6 months. He tolerated therapy well, with complete resolution of abdominal pain and no new gastrointestinal symptoms. A follow-up contrast-enhanced computerized tomography scan (Fig. 2) showed a dramatic reduction in the size of the hepatic lesions and resolution of cecal wall thickening, indicating a marked radiologic and clinical response to antifungal treatment.

### Case 2

A 14-year-old girl was referred to the hospital after being discharged against medical advice from a local facility. She had a 6-month history of markedly worsening chronic diarrhea, with ˃10 mucous-laden stools per day, associated with recurrent nausea and vomiting containing food particles and occasionally blood, and an unintentional weight loss of ~20 kg over the preceding year. Her mother reported that the patient had experienced intermittent mucous diarrhea since infancy. Additional symptoms included recurrent oral ulcers and occasional palpitations; there was no history of headache, visual disturbance, dysphagia, chest pain, dyspnea, dysuria, or change in urinary habits.

At the local hospital, she had been labeled as having ulcerative colitis based solely on colonoscopic impressions of perianal erosions and ulcerations on digital rectal examination and pressure-induced edematous changes of the rectal and sigmoid walls with pale mucosa but no erythema or definite ulceration. Histology did not confirm inflammatory bowel disease, yet mesalazine therapy was commenced. During that admission, she developed severe hypokalemia complicated by cardiac arrest, requiring prolonged intensive care admission with subsequent residual neurological deficits.

**Figure 3:**
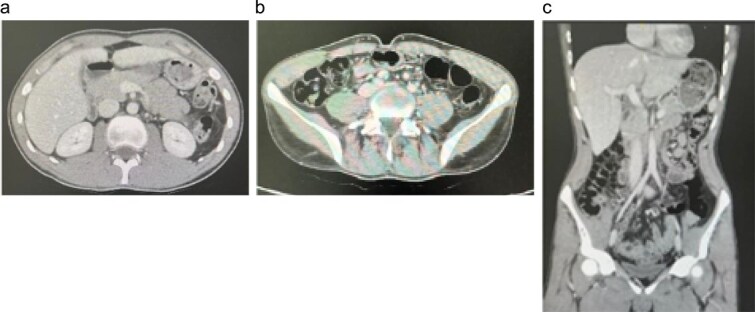
Follow-up contrast-enhanced CT abdomen in Case 1. (a) Axial image through the upper abdomen showing interval disappearance of the previously described hepatic lesions. (b) Axial image at the level of the right iliac fossa demonstrating resolution of cecal/right colonic wall thickening and surrounding inflammatory changes. (c) Coronal reformatted image confirming normalization of right colon and liver appearance, consistent with an excellent radiologic response to prolonged voriconazole therapy.

On presentation to our center, she appeared cachectic with persistent neurological impairment. Abdominal examination revealed a soft, lax abdomen without localized tenderness or palpable masses. Laboratory investigations showed systemic inflammation and anemia, with an erythrocyte sedimentation rate of 56 mm/h, a white blood cell count of 13.5 × 10^9^/L, a C-reactive protein of 205 mg/L, and a hemoglobin of 8.9 g/dL. Abdominal MRI demonstrated extensive circumferential thickening of the rectum and sigmoid colon with widespread peritoneal disease, bilateral mild hydronephrosis, and a loculated fluid collection in the upper pelvis (Fig. 3). The differential diagnosis included atypical infections such as tuberculosis and fungal disease (basidiobolomycosis or actinomycosis), as well as adenocarcinoma and lymphoma. Tumor markers and microbiological studies were negative.

**Figure 4:**
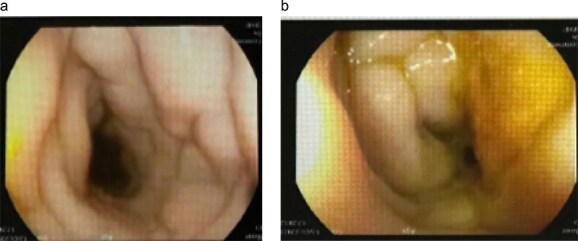
Colonoscopic finding. (a) Colonoscopic views of the rectosigmoid colon demonstrate marked concentric narrowing with pale, edematous mucosal folds and loss of regular vascular pattern, resulting in near-luminal occlusion. (b) These appearances were initially suggestive of severe inflammatory or infiltrative disease and were subsequently proven to represent GIB.

Image-guided biopsy from the omentum showed non-necrotizing granulomatous inflammation with broad, aseptate fungal hyphae consistent with basidiobolomycosis. Colonoscopy demonstrated a markedly narrowed, inflamed rectosigmoid segment (Fig. 4).

**Figure 5:**
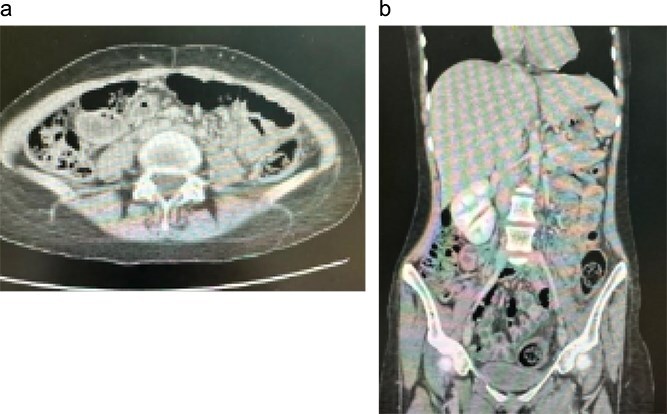
Follow-up contrast-enhanced CT abdomen and pelvis. (a) Axial image demonstrating resolution of the previously noted circumferential rectosigmoid wall thickening and pericolic inflammatory changes, with no residual pelvic mass or collection. (b) Coronal reformatted image confirming marked regression of rectosigmoid and peritoneal disease, consistent with a favorable radiologic response to prolonged voriconazole therapy.

The patient was commenced on intravenous voriconazole 200 mg twice daily on 26 June 2023 and was transitioned to oral voriconazole 200 mg twice daily in January 2024 for a planned 1-year course. She exhibited marked clinical and radiologic improvement, with follow-up computerized tomography scan shown in Fig. 5, showing regression of rectosigmoid and peritoneal disease, and was discharged home with planned outpatient follow-up. Subsequent surveillance imaging demonstrated recurrence of rectosigmoid and pelvic disease; however, she remains clinically asymptomatic and continues oral voriconazole under close multidisciplinary review to determine the need for treatment escalation or additional therapeutic interventions.

She was discharged home in stable condition. Subsequent follow-up imaging demonstrated recurrence of rectosigmoid and pelvic disease; however, she remains free of gastrointestinal symptoms and continues on oral voriconazole under close multidisciplinary follow-up to assess the need for treatment escalation or adjunctive interventions.

## Discussion

The cases from this tertiary centre show that GIB in immunocompetent patients can present as an apparent right-sided colonic malignancy with hepatic lesions or as severe, refractory rectosigmoid colitis with peritoneal disease. In both, deep tissue histology (cecal mass biopsy and image-guided omental biopsy) demonstrated granulomatous inflammation with broad, sparsely septate fungal hyphae, prompting reclassification of the pathology and initiation of voriconazole, with substantial clinical and radiologic improvement and preservation of bowel continuity.

Published reviews and case series describe GIB in immunocompetent hosts, often masquerading as colorectal cancer, inflammatory bowel disease or abdominal tuberculosis [2, 9]. The cecal mass with hepatic lesions and the segmental rectosigmoid colitis in our patients mirror the imaging and endoscopic patterns reported in recent multicase descriptions [8]. Voriconazole-based, organ-sparing management is consistent with reports in which azole monotherapy has achieved cure without primary bowel resection in selected patients [13, 14]. *Basidiobolus ranarum* is an environmental saprophyte found in soil and the gastrointestinal tracts of amphibians and reptiles; ingestion of contaminated material is considered the main route of human infection [2]. After colonization of the bowel wall, the fungus elicits a transmural granulomatous, often eosinophil-rich inflammatory response that produces mass-forming lesions and adjacent inflammatory change [15, 16]. The age and immune status of our patients fit this profile and support the concept that GIB should be considered even in otherwise healthy hosts [2].

Diagnosis was challenging because laboratory tests and cross-sectional imaging were non-specific and initially suggested malignancy or chronic inflammatory bowel disease. As shown in previous series, superficial mucosal biopsies may miss disease that predominantly involves the submucosa and deeper layers [17]. Targeted cecal mass biopsy and image-guided omental biopsy yielded diagnostic tissue and avoided immediate radical surgery [10]. Voriconazole was selected in the absence of obstruction or perforation and produced a sustained radiologic response, consistent with published experience with triazoles as primary therapy [13].

These observations underscore that GIB should be considered when young immunocompetent patients present with a colonic mass and hepatic lesions, or with refractory colitis and peritoneal thickening, particularly when histology is not typical of carcinoma or inflammatory bowel disease [10]. Surgeons indicate that histologic confirmation, when feasible, can prevent unnecessary radical colorectal resection. A multidisciplinary discussion guided the decision to pursue medical rather than extensive surgical management.

Strengths of this report include detailed clinicoradiologic and histopathologic descriptions, as well as serial imaging documenting the response to antifungal therapy. Significant limitations include the small number of patients from a single centre and the absence of culture or molecular data in the present account, which restricts strain-level characterization and correlation with antifungal susceptibility. Follow-up is relatively short, so the optimal treatment duration and long-term relapse risk remain uncertain.

## Conclusion

GIB is an uncommon but important cause of mass-forming and infiltrative colonic and peritoneal disease in immunocompetent hosts. In this single-center series of three patients, presentations included apparent right-sided colonic malignancy with hepatic lesions and severe rectosigmoid colitis with extensive peritoneal involvement, all initially considered inflammatory or neoplastic conditions. Definitive diagnosis was achieved only after deep tissue sampling and histopathological demonstration of granulomatous inflammation with broad pauciseptate fungal hyphae consistent with *B. ranarum*. Early recognition of this pattern, together with a high index of suspicion in appropriate epidemiological settings, can prevent inappropriate immunosuppressive or oncologic treatments and facilitate timely initiation of antifungal therapy. Prolonged voriconazole therapy led to substantial clinical and radiologic improvement. It allowed avoidance of radical colorectal surgery in all patients, although radiologic recurrence in one case underscores the need for careful long-term follow-up and individualized treatment duration. Clinicians and surgeons should therefore include GIB in the differential diagnosis of unexplained segmental colitis, colonic masses, and peritoneal thickening in immunocompetent patients.

## Highlights

- Gastrointestinal basidiobolomycosis is a rare fungal infection that clinically mimics colorectal malignancy or inflammatory bowel disease, leading to delayed recognition in both cases.
- Routine endoscopic biopsies were non-diagnostic, and a definitive diagnosis required deep-tissue sampling showing granulomatous inflammation with fungal elements.
- Early initiation of voriconazole led to marked clinical improvement, and both patients were successfully managed without surgical resection.
- Follow-up imaging demonstrated progressive resolution of bowel wall thickening and masses, confirming effective response to antifungal therapy alone.
- These cases highlight the need to consider fungal etiologies in unexplained colonic masses or refractory colitis, particularly in endemic regions.
